# The Guardian of Health: A Monthly Journal of Domestic Hygiene

**Published:** 1841-11-06

**Authors:** 


### BIBLIOGRAPHICAL NOTICES.

*The Guardian of Health: a monthly Journal of Domestic Hygiene.* Edited by Thomas E. Bond, Jr., M. D., and Chapin A. Harris, M. D. Nos. 1, 2, and 3—September, October, and November, 1841. Baltimore: Knight & Colburn.

We find upon our table a fasciculus of forty-eight pages, bearing this title. A well conducted journal, published with the view of enlightening the public upon hygienic principles, is a desideratum. The early and great success of the Journal of Health, formerly published in this city, proved the popularity of the subject, and, for a time, was productive of much good. It died, we fear, in part, from a seeming exhaustion of the subject; and, indeed, this must be the fate of any such undertaking in the course of a few years, unless a wider scope be given to the enterprise than is usually intended by those who aim at an audience beyond the circle of the scientific. The Annales d'Hygiène Publique, of which the twenty-fifth volume lies beside the little Baltimorean publication, is addressed to the profoundly learned, and contains articles of great weight and importance; but most of the periodicals designed for *the general reader* have been limited in duration to the completion of two or three volumes. They are prone to become insufferably heavy on the one hand, or to sink into mere twaddle on the other. We sincerely hope that the Guardian of Health may prove more fortunate; but to do so, it must secure the aid of high talent, and eschew all trifling articles, such as are too frequently forced into the columns of a journal by the disagreeableness of refusal, or the fear of offending a subscriber. The number or numbers before us contain several well-selected articles, and take high and just ground against empiricism, and also against some almost intangible absurdities of the day, that will not willingly consent to receive that title. Among the rest, it contains an extract from the Boston Medical and Surgical Journal on Homœopathy, with a most amusing postscript, in which the *numerical system* is brought to bear on the question of the quantity of fluid required properly to dilute one drop of a tincture to render it *homœopathically efficient.* The calculation is stopped in mid career by the inability of the page to accommodate the line of figures; but it is made very evident that the distance *from the earth to the nearest fixed star* would be a tolerably good measure of the side of a cube large enough to contain the mixture! What infinite expansion of imagination, and what *infinitesmal force of intellect*, must be required to grasp *the stupendous nothingness* of this all-conquering theory! But it is folly to frown where smiles are more appropriate, and there are weaknesses which call for pity rather than anger. In applying the terms "dishonest and unprincipled" to the sect of homœopaths, the correspondent of the Boston Journal has been guilty of injustice, and the editor of the Guardian of Health did wrong to endorse the charge. These terms are applicable only to those who *profess* the doctrines of the sect, while they *practise* an opposite course, in order to play upon the prejudices of the ignorant. We have known some very amiable and exceedingly good-natured people, regularly enrolled on the lists of the profession, who fully believed in the truth of what is called by them the doctrine of homœopathy; nor can it be maintained, even by their antagonists, that *they* are not safer, and society better served, when they act up to their principles, than if they were to pursue an opposite course.

Again—Many have grown wiser by experience, and have relinquished the doctrine that *the half is greater than the whole*, and only contend that *the best way to make a man better, is to make him a little worse!*

Since we are in a facetious mood, it may not be amiss to say, in the utmost good humour, (and we know the author will excuse the raillery,) that one phrase in the "Dissertation on the Management of the Mouth and Teeth, by L. S. Parmley, M. D., D. D. S., reminds us of the ancient proposition *to fortify a city with leather!*

"This false doctrine of hereditary weakness" (of the teeth) "appears to have but one sure, unerring remedy; and that is, to familiarize mothers, guardians and teachers, with *the patent apparatus I have invented*," &c. p. 23

As the article from which the above quotation is made throws a curious light upon the infinitude of evil resulting from the neglect of teeth, both in childhood and adult age,—evils, some of which some of us have been in the habit of attributing to other causes,—we select the following passage to instruct our readers in the proper method of preserving those all-important organs. The process is to commence in childhood, "soon after the fifth year," when the four permanent molar teeth appear.

"This class of double teeth, intended to receive the whole pressure and force of the jaws during the changing of all the front teeth, the most critical epoch in life, therefore the same clean habit should be faithfully continued, as particular daily attention will prevent irreparable mischief, supersede the ordinary services of the dentist, who should inspect the mouth (or some qualified person) once a month, and then the daily care of cleaning it perfectly after the principal meals with the following instruments, appear indispensable for comfort and safety. First, a stiff brush of a suitable size, used freely over the crowns of the teeth, and moved briskly up and down as may be convenient to give friction to the gums at the same time. Secondly, The floss silk well waxed should be passed up and down on the right and left side of each tooth, a little under the gums, which will remove all accumulations from the interstices and necks of the teeth, where the brush cannot reach. Thirdly, The tooth-polisher, formed so as to be applied to the surface of the teeth where any discoloration may be seen, and near the gums or edges; the friction to be continued until the enamel is smooth. Fourthly, The double instrument for removing hard tartar, and separating the gums during the growth of the teeth, so as to make room to polish and clean the enamel thus exposed, and where the ordinary means cannot be applied with good effect. Fifthly, The tongue scraper, of whalebone, (well made,) should be used every morning, at least, to free the surface and edges of the tongue from foul deposites, and discharge the glands, and remove all secretions from the mouth, which would injure its general health, and prove detrimental to the stomach and lungs of the most vigorous temperament at any period of life. If these instructions are properly persevered in, and the means faithfully used, the gums and teeth of every shape and colour may be preserved through life by every class of people, with little expense, in all climates, and the breath from taint, a consummation devoutly to be wished."

Our chief fear as to the above directions—if intelligible—is, that amidst the many cares of life, the public will be unwilling to devote the necessary time to this important business, or that other hygienic operations of daily necessity may be unduly neglected therefor.

In taking leave of this new candidate for patronage, it is but right to state that its pages *really* contain a fair proportion of matter of high practical value, and most of the articles are chosen with the evident design to combat existing prejudices, and diffuse knowledge of practical value among a class of readers who are very much in want of it. So long as the Journal avoids the two great dangers already pointed out, it will flourish; and so long as it supports the standard of *anti-empiricism*, we shall ardently desire its success.

